# Comorbidity network analysis related to obesity in middle-aged and older adults: findings from Korean population-based survey data

**DOI:** 10.4178/epih.e2021018

**Published:** 2021-03-05

**Authors:** Hye Ah Lee, Hyesook Park

**Affiliations:** 1 Clinical Trial Center, Ewha Womans University Mokdong Hospital, Seoul, Korea; 2 Department of Preventive Medicine, Ewha Womans University College of Medicine, Seoul, Korea; 3 Graduate Program in System Health Science and Engineering, Ewha Womans University, Seoul, Korea

**Keywords:** Comorbidity, Multimorbidity, Obesity, Network analysis

## Abstract

**OBJECTIVES:**

We conducted a comorbidity network analysis using data from the seventh Korea National Health and Nutrition Examination Survey to systematically quantify obesity-related comorbidities.

**METHODS:**

The study included 11,712 subjects aged 45 to 80 (5,075 male and 6,637 female). A prevalent disease was defined as a specific disease for which a subject had been diagnosed by a doctor and was being treated. Comorbidity network analysis was performed for diseases with a prevalence of 1% or more, including overweight and obesity. We estimated the observed-to-expected ratio of all possible disease pairs with comorbidity strength and visualized the network of obesity-related comorbidities.

**RESULTS:**

In subjects over 45 years old, 37.3% of people had a body mass index over 25.0 kg/m^2^. The most common prevalent disease was hypertension (42.3%), followed by dyslipidemia (17.4%) and diabetes (17.0%). Overweight and obese subjects were 2.1 times (95% confidence interval, 1.9 to 2.3) more likely to have a comorbidity (i.e., 2 or more diseases) than normal-weight subjects. Metabolic diseases such as hypertension, dyslipidemia, diabetes, and osteoarthritis were directly associated with overweight and obesity. The probability of coexistence for each of those 4 diseases was 1.3 times higher than expected. In addition, hypertension and dyslipidemia frequently coexisted in overweight and obese female along with other diseases. In obese male, dyslipidemia and diabetes were the major diseases in the comorbidity network.

**CONCLUSIONS:**

Our results provide evidence justifying the management of metabolic components in obese individuals. In addition, our results will help prioritize interventions for comorbidity reduction as a public health goal.

## INTRODUCTION

According to a World Health Organization report, 39% of adults over the age of 18 worldwide had a body mass index (BMI) of 25.0 kg/m^2^ or higher in 2016 [1]. This trend has increasing steadily over the past 3 decades [2], and a similar trend has been observed in Korea [3]. The high prevalence of obesity is a public health challenge. In 2017, the Global Burden of Disease project reported that high BMI accounted for 4 million deaths globally and also contributed to 120 million disability-adjusted life-years [4]. As normal weight maintenance and weight loss are associated with many health benefits, obesity prevention and management are among the top public health priorities. Moreover, the global prevalence of overweight and obesity among children and adolescents aged 5 to 19 increased from 4% in 1975 to 18% in 2016 [1]. Due to this trend, the burden of obesity is expected to continue.

It is well-known that obesity is a major risk factor for chronic diseases such as cardiovascular disease (CVD), some type of cancers, and metabolic diseases [5]. While obesity is clearly linked to a variety of diseases, there continue to be questions about how many obesity-related diseases exist and how closely they relate to each other. The relationship between various diseases and obesity is not straightforward and can be difficult to explain. For example, the effect of obesity on CVD could be explained by other mediating metabolic diseases. Although some studies have shown relationships between obesity and various diseases [6,7], few have quantified these relationships overall. In order to quantify these relationships, we conducted a comorbidity network analysis. Network analysis is a methodological approach for the systematic evaluation of complex relationships [8] that can help illuminate the natural progression of chronic diseases. Studies on diabetes and hypertension have reported results of comorbidity network analyses based on claims data [9,10]. Since obesity is not classified as a clinical disease, it is only possible to grasp the relationship between obesity and other diseases using large-scale survey data, rather than administrative data. Therefore, in this study, we systematically analyzed comorbidity patterns related to obesity through network analysis using data from the Korea National Health and Nutrition Examination Survey (KNHANES).

## MATERIALS AND METHODS

### Data

For this study, we used data from the seventh KNHANES (2016- 2018). The KNHANES is a nationally representative survey that is conducted annually to assess health-related behaviors, chronic disease conditions, and trends in food and nutrition intake. The KNHANES consists of 3 component surveys: a health interview, a health examination, and a nutrition survey. A sampling plan was conducted before every wave of the survey (every 3 years) and relied on a multi-stage sampling method. The detailed KNHANES survey method has been published elsewhere [11]. The seventh survey collected data from 2016 to 2018, with a response rate of 76.6%. It collected data on 24,269 people between 1 year and 80 years old (8,150 in 2016, 8,127 in 2017, and 7,992 in 2018). Since the prevalence of chronic diseases increases with age, we only included middle-aged and elderly subjects (45-80 years old, n= 12,193). After the exclusion of subjects with missing data regarding disease-related questions and BMI, 11,712 subjects were ultimately included in the study (5,075 male and 6,637 female).

### Overweight and obesity definition and assessment of prevalent diseases

A person with a BMI equal to or more than 25.0 kg/m^2^ was considered overweight/obese. Overweight and obesity were determined based on BMI, which was calculated using data from measurements of subjects’ height and weight. Although the criterion for overweight in Asian countries is lower than that in Western countries, this study used the criterion of 25.0 kg/m^2^ as a general standard to define overweight. Thirty diseases were investigated using data from the seventh KNHANES survey of chronic disease conditions. They were hypertension, dyslipidemia, stroke, coronary artery disease (myocardial infarction or angina), osteoarthritis, rheumatoid arthritis, osteoporosis arthritis, tuberculosis, asthma, diabetes, thyroid disease, stomach cancer, liver cancer, colorectal cancer, breast cancer, cervical cancer, lung cancer, thyroid cancer, depression, atopic dermatitis, allergic rhinitis, sinusitis, otitis media, cataract, glaucoma, macular degeneration, kidney failure, hepatitis B, hepatitis C, and cirrhosis. A prevalent disease was defined as a specific disease in a subject that had been diagnosed by a doctor and was being treated at the time of the survey. Hypertension and diabetes specifically were determined using blood pressure and fasting blood glucose data according to the criteria reported by the Korea Centers for Disease Control and Prevention [12,13]. These variable units were labeled 0 (no) and 1 (yes).

### Comorbidity network analysis

The research goal of the comorbidity network was to assess whether diseases tend to appear together more frequently than expected. The basic elements of network analysis consist of nodes and edges. Nodes indicate specific diseases, and edges indicate the coexistence of a pair of diseases. Due to the nature of the study design, the edges between diseases have no directionality. To identify obesity-related comorbidities, we considered overweight and obesity (BMI≥ 25.0 kg/m^2^) as a node. For comorbidity strength, we estimated the observed-to-expected ratio (OER) of all possible disease pairs based on previous studies of comorbidity networks [14]. The numerator of the OER is the observed coexistence scale of a disease pair, and the denominator is the expected coexistence scale of a disease pair under the assumption of independence. Thus, the OER can be expressed as follows:

$$OER_{i,j}=\frac{a}{b\times c}$$

Where *a* is the proportion with coexistence of diseases *i* and *j*; *b* is the prevalence of disease *i*; *c* is the prevalence of disease *j*.

Statistical tests were performed on the proportional differences between the denominator and the numerator, and p-values were calculated with continuity correction. For disease pairs with pvalue< 0.05 and OER> 1.0, the relationship was visualized using visNetwork, an R package for network visualization. The diameter of each node represents the prevalence of each disease, and the thickness of edges represents the OER value. Network clustering was analyzed using the “infomap” algorithm of the igraph R package [15]. Thus, the same clusters are represented by nodes of the same color in our visualizations. We estimated the strength, degree, closeness, and betweenness of diseases (nodes) in the network to determine their relative importance. The strength is determined by the sum of the strength of all the connections of a specific disease. Degree is measured by the number of direct connections to a disease. Closeness is a quantified relationship that includes indirect connections from a specific disease in the network. High closeness, for example, indicates a short average distance between a specific disease and all other diseases. Betweenness measures the importance of nodes in the average pathway between other pairs of diseases [8]. For every pair of diseases in a connected graph, there is at least 1 shortest path between diseases. The betweenness of each disease is defined as the number of these shortest paths going through a disease. Diseases present in less than 1% of the total population were not included in the network analysis. We performed an initial comorbidity network analysis including all subjects, followed by analyses of people considered obese of each sex. A sensitivity analysis was performed with overweight and obesity defined using a BMI threshold of 23.0 kg/m^2^ or higher.

### Statistical analysis

For summary statistics, the results for continuous variables were presented as weighted means with standard error (SE), while those for categorical variables were presented as the number of subjects with weighted percentages based on multi-stage sampling. The 30 diseases were listed according to the prevalence rate, and diseases with a prevalence rate of 1% or more were tabulated. The risk of having a prevalent disease according to sex or obesity was evaluated using logistic regression and results were expressed as an odds ratio (OR) with a 95% confidence interval (CI). Statistical significance was assessed at p-value < 0.05 with a 2-tailed test. All statistical analyses were performed using SAS version 9.4 (SAS Institute Inc., Cary, NC, USA) or R version 3.6.2 (https://cran.rproject.org/bin/windows/base/old/3.6.2/).

### Ethics statement

There was no personal information included in the KNHANES data, and ethical approval for the use of open KNHANES data was exempted from the Institutional Review Board (IRB) Committee of the Ewha Womans University Hospital (IRB No. EUMC 2020-08-007).

## RESULTS

Of subjects over 45 years old, 37.33% (SE, 0.56) had a BMI that exceeded 25 kg/m^2^. Of that percentage, female accounted for 49.16% (SE, 0.88). It was reported that 70.43% (SE, 0.84) of obese subjects had at least 1 of the 30 prevalent diseases, and 40.16% (SE 0.86) had 2 or more prevalent diseases. In non-obese subjects, 56.38% (SE, 0.75) of subjects had 1 prevalent disease, and 26.63% (SE, 0.65) had 2 or more prevalent diseases (Table 1).

The associations between high BMI (≥ 25.0 kg/m^2^) and having a disease with a prevalence rate of more than 1% are presented in Table 2. The most common disease was hypertension (42.28%), followed by dyslipidemia (17.43%) and diabetes (17.04%). Those with overweight and obesity had a 2.34 times (95% CI, 2.12 to 2.58) higher risk of hypertension than those who are not obese. Overweight and obesity were also associated with prevalent diseases such as dyslipidemia, diabetes, osteoarthritis, thyroid disease, stroke, and coronary artery disease. The high-BMI group had a 2.08 times (95% CI, 1.89 to 2.29) higher risk of comorbid disease than their low-BMI counterparts when adjusted for sex and age.

**Figure 1.**
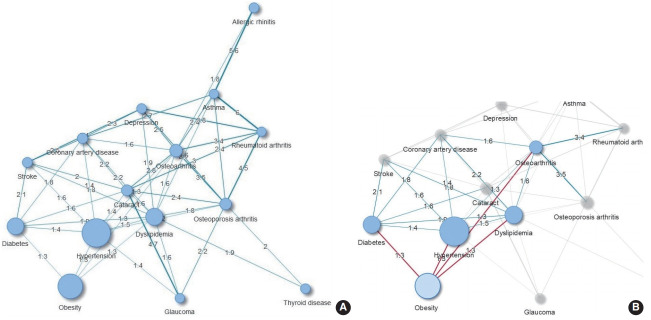
Comorbidity network in adults over 45 years of age (A) and obesity-related comorbidities (B) (n=11,712). Node size is proportional to the prevalence of the corresponding comorbidity. Same-colored nodes indicate the same cluster. Edge thickness indicates the observed-to-expected ratio value for connected conditions.

The results of the comorbidity network analysis related to obesity are shown in Figure 1. One cluster was identified (Figure 1A). The number of subjects with obesity who also had hypertension, diabetes, dyslipidemia, or osteoarthritis was 30% higher than expected. Additionally, obesity was indirectly associated with CVDs through metabolic diseases (hypertension, diabetes, and dyslipidemia). Cataracts were linked to obesity through diabetes (Figure 1B). The results were also consistent when defining overweight and obesity as having a BMI of 23.0 kg/m^2^ or higher (data not shown).

We also evaluated the rates of prevalent diseases in obese subjects by sex (Table 3). Dyslipidemia and musculoskeletal diseases were more common in females than in males. Cataracts, thyroid disease, and depression were also more frequent in females than in males. However, hypertension, diabetes, and coronary artery disease were more common in males than in females. In overweight and obese subjects, the comorbidity risk was higher in females than in males by 1.22 times (95% CI, 1.05 to 1.42) after the results were adjusted for age.

**Figure 2.**
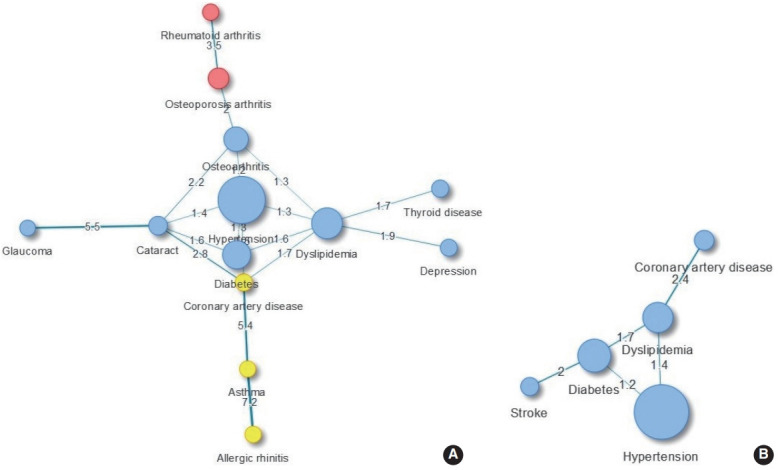
Comorbidity network in obese female (A) and obese male (B) over 45 years of age. Node size is proportional to the prevalence of the corresponding comorbidity. Same-colored nodes indicate the same cluster. Edge thickness indicates the observed-to-expected ratio value for connected conditions.

We evaluated the major comorbidities of obese subjects by sex using network analysis. In females, 3 clusters were identified: (1) metabolic diseases, thyroid disease, depression, and ophthalmic diseases were classified into one cluster; (2) musculoskeletal diseases; (3) otorhinolaryngologic and coronary artery diseases. Diseases such as thyroid disease and depression were shown to accompany dyslipidemia, and the proportion of diabetes and cataract comorbidities was 1.64 times as high as expected (95% CI, 1.09 to 2.47) (Figure 2A). In obese males, the proportion of dyslipidemia and coronary artery disease comorbidities, which appeared as a cluster, was more than 2.4 times higher than expected, and the proportion of diabetes and stroke comorbidities was 2 times higher than expected (Figure 2B). When the comorbidity network was analyzed for betweenness, hypertension and dyslipidemia were the major diseases in obese females, and dyslipidemia and diabetes were the major diseases in obese males. These tendencies remained unchanged even when an analysis based on closeness was conducted (Table 4).

## DISCUSSION

Through comorbidity network analysis, we identified the major obesity-related comorbid diseases using large-scale survey data. Metabolic diseases such as hypertension, dyslipidemia, diabetes, and osteoarthritis were directly associated with overweight and obesity, and the comorbidity risk in overweight and obesity subjects was 2.1 times higher than in normal-weight subjects. Furthermore, in overweight and obese people, dyslipidemia, hypertension, and diabetes were frequently accompanied by other diseases, and were characterized as close to other diseases. Moreover, based on their betweenness, we found that these metabolic diseases play a central role in mediating between diseases in the network. Therefore, managing metabolic components and weight loss reduces the likelihood of developing BMI-related diseases and comorbidities.

Although obesity has been accepted as a predisposing factor for the development of various chronic diseases such as CVD and metabolic diseases, the definition of obesity-related diseases has unclear boundaries. Comorbidity network analysis provides insights to help understand obesity-related diseases and can provide information for patient care and treatment in a medical setting [9,10,16]. By obtaining data on the prevalence of chronic diseases with a long disease duration, a snapshot was taken of the distribution of comorbid diseases, and our results are in line with those of previous studies. Some paired diseases had a higher prevalence than expected in terms of the OER. This finding means that these diseases do not have an independent distribution, but instead are closely related to other conditions.

The relationship between metabolic diseases and high BMI is well established based on accumulated evidence [5,7]. Large-scale survey studies in the United States and Europe have reported that hypertension, dyslipidemia, and diabetes are linked to overweight and obesity in a dose-dependent manner [17,18]. Among the 4 diseases directly related to overweight and obesity, metabolic diseases are frequently accompanied by other diseases, and CVD, which is significantly related to obesity, appears to have a relationship to these diseases. A pathway mediated by metabolic diseases in the relationship between obesity and CVD has also been suggested in previous studies [5,19]. Coronary artery disease has been shown to accompany dyslipidemia in both obese males and females, and was 2.4 times and 1.7 times more frequent than expected, respectively. The coexistences of asthma and coronary artery disease was significant in obese females. According to a metaanalysis, an association between asthma and coronary artery disease was predominantly found in females [20]. In addition, obesity had a high likelihood of being accompanied by hypertension, diabetes, and dyslipidemia (p-value for all pairs < 0.01) (Figure 2), which may increase the risk of subsequent disease. Indeed, a study found that the prevalence of coronary artery disease was often more than doubled among patients with concomitant conditions with hypertension and dyslipidemia compared to those with only 1 condition [21]. Therefore, it is important for clinicians to understand and manage comorbid diseases in order to prevent diseases that increase patients’ risk of disability and premature death.

The prevalence of musculoskeletal diseases was higher in obese females than in obese males, and the coexistence of osteoarthritis and hypertension was also found to be significant in obese females, which aligns with the results of previous studies [22-24]. The relationship between osteoarthritis and hypertension remains controversial, but it has been proposed that obesity may be involved as a pathomechanism [25]. Musculoskeletal diseases are known to impair functional parameters such as mobility, subsequently reducing quality of life [26]. A comorbidity study showed that those with osteoarthritis reported a low quality of life regardless of the type of comorbid disease [23]. A quantitative systematic review evaluated the effect of weight loss on osteoarthritis symptoms and reported that weight loss in obese patients diagnosed with knee arthritis appeared to improve physical disability and pain levels [27]. Since the likelihood of musculoskeletal diseases increases with age, efforts to improve quality of life are needed along with treatment and management.

Among endocrine diseases, thyroid disease was significantly associated with overweight and obesity. Although the types of thyroid diseases associated with overweight and obesity have not been investigated in detail, a study in Korea showed that hypothyroidism has a higher prevalence than hyperthyroidism [28]. Of these diseases, hypothyroidism is more closely related to obesity, and it may also affect lipid profiles [29]. In an UpToDate review, it was reported that patients with hypercholesterolemia should be screened for hypothyroidism before lipid-lowering therapy [30]. However, since thyroid disease is underdiagnosed, the accuracy of the assessment of the coexistence of thyroid disease with other diseases seems to be low. Among other endocrine diseases, diabetes commonly coexisted with hypertension or dyslipidemia, but it also coexisted frequently with cataracts. These results are consistent with those of a previous study [10]. The coexistence of diabetes and cataracts and the coexistence of thyroid disease and dyslipidemia were more prevalent in obese females than in obese males. On the contrary, the coexistence of stroke and diabetes was frequent in obese males, but not in obese females. It seems that there were meaningful differences in the prevalence of these conditions between male and female. Sex differences in the prevalence of comorbidities have been studied elsewhere and help to account for differences in life expectancy between male and female [18,31].

There were several points to keep in mind when interpreting the results. Our results were derived from a cross-sectional survey, so an analysis of causality is not appropriate. Some uncertainty by measurement error and self-reported disease information may have influenced the results. The data source used did not include patients with severe conditions who had been hospitalized, which would have affected prevalence estimates. Our study utilized measured BMI information, but the diseases that could be considered were limited according to the scope of the survey data. Although a positive association between obesity and non-alcoholic fatty liver disease (NAFLD) has been reported [5], NAFLD is not covered in the KNHANES. Using a limited range of survey data inevitably limited the scope of our evaluation. While we used 3-year combined data, the data used for our analysis were utilized on a small scale according to the conditions of interest. Therefore, we were limited in our ability to subdivide subjects into separate overweight and obese groups. In addition, since significance was determined according to the size of the dataset, the significance level was not controlled for multiple comparisons.

Nevertheless, results were obtained using data representing the Korean population, so there are benefits to the generalized scope of the results. By using survey data rather than administrative data, our study provides information on obesity-related comorbidities through a systematic approach. Its findings can motivate the public to manage and prevent, as well as better understand, overweight and obesity. Finally, through network analysis, we systematically evaluated obesity-related diseases and provided insights for further research. The study can also provide helpful information for prioritizing interventions for comorbidity reduction as a public health measure.

In summary, taking into account the possibility of modification, preventing overweight and obesity through weight loss is expected to reduce the risk and severity of various chronic diseases. Furthermore, managing metabolic components in obese persons reduces the chance of developing comorbid diseases.

## Figures and Tables

**Table 1.** Basic characteristics of study subjects

Characteristics	BMI <25.0 kg/m^2^ (n=7,356)	BMI ≥25.0 kg/m^2^ (n=4,356)
Age (yr)	59.73 [0.18]	59.60 [0.22]
Sex, male	3,111 (45.94)/[0.59]	1,964 (50.84)/[0.88]
BMI (kg/m^2^)	22.29 [0.03]	27.44 [0.04]
No. of comorbidities among 30 prevalent diseases		
0	2,889 (43.62)/[0.75]	1,111 (29.57)/[0.84]
1	2,187 (29.75)/[0.64]	1,293 (30.28)/[0.79]
2	1,328 (15.86)/[0.49]	1,034 (22.33)/[0.75]
3	610 (7.15)/[0.35]	562 (11.11)/[0.50]
4	235 (2.50)/[0.20]	246 (4.74)/[0.37]
≥5	107 (1.11)/[0.13]	110 (1.98)/[0.22]

Values are presented as mean [standard error] or number (weighted %)/[standard error]. BMI, body mass index.

**Table 2.** Diseases with a prevalence of more than 1% in adults over 45 years of age and differences according to high BMI (n=11,712)^1^

Ranking of prevalent diseases	Total (n=11,712)	BMI, kg/m^2^		
		<25.0 (n=7,356)	≥25.0 (n=4,356)	≥25.0 vs. <25.0^2^
1. Hypertension	5,362 (42.28)	2,861 (35.41)	2,501 (53.83)	2.34 (2.12, 2.58)
2. Dyslipidemia	2,274 (17.43)	1,182 (14.22)	1,092 (22.81)	1.84 (1.65, 2.06)
3. Diabetes	2,173 (17.04)	1,115 (13.78)	1,058 (22.51)	1.87 (1.68, 2.08)
4. Osteoarthritis	1020 (7.20)	529 (5.91)	491 (9.36)	1.69 (1.44, 1.99)
5. Osteoporosis arthritis	760 (5.26)	505 (5.45)	255 (4.93)	0.83 (0.69, 1.01)
6. Cataracts	573 (3.89)	370 (4.10)	203 (3.54)	0.84 (0.68, 1.04)
7. Coronary artery disease	444 (3.09)	249 (2.74)	195 (3.68)	1.44 (1.16, 1.79)
8. Allergic rhinitis	325 (2.90)	211 (3.02)	114 (2.69)	0.90 (0.68, 1.18)
9. Stroke	301 (2.26)	176 (2.04)	125 (2.63)	1.35 (1.01, 1.79)
10. Thyroid	266 (2.17)	154 (1.96)	112 (2.52)	1.41 (1.04, 1.90)
11. Depression	255 (1.86)	152 (1.77)	103 (2.01)	1.15 (0.86, 1.54)
12. Asthma	175 (1.31)	105 (1.26)	70 (1.38)	1.10 (0.78, 1.57)
13. Glaucoma	181 (1.29)	111 (1.20)	70 (1.43)	1.23 (0.87, 1.74)
14. Rheumatoid arthritis	157 (1.17)	93 (1.20)	64 (1.11)	0.93 (0.64, 1.36)
Comorbidity (2 or more diseases)^3^	4,232 (31.68)	2,280 (26.63)	1,952 (40.16)	2.08 (1.89, 2.29)

Values are presented as number (weighted %) or odds ratio (95% confidence interval). BMI, body mass index.

1 The results were obtained taking into account the sampling design.

2 Adjusting for sex and age, taking people with a BMI <25.0 kg/m^2^ as the reference group.

3 Comorbidity was defined as people with 2 or more of the 30 diseases.

**Table 3.** Diseases with a prevalence of more than 1% in overweight and obese adults over 45 years of age by sex^1^

Ranking of prevalent diseases	Total (n=4,356)	Male (n=1,964, 45.1%)	Female (n=2,392, 54.9%)	Female vs. male^2^
1. Hypertension	2,501 (53.83)	1,123 (54.13)	1,378 (53.52)	0.70 (0.60, 0.81)
2. Dyslipidemia	1,092 (22.81)	414 (19.20)	678 (26.55)	1.26 (1.07, 1.49)
3. Diabetes	1,058 (22.51)	494 (22.34)	564 (22.69)	0.81 (0.69, 0.97)
4. Osteoarthritis	491 (9.36)	85 (3.51)	406 (15.41)	3.87 (2.90, 5.18)
5. Osteoporosis arthritis	255 (4.93)	9 (0.38)	246 (9.63)	20.05 (9.04, 44.45)
6. Cataracts	203 (3.54)	48 (1.67)	155 (5.48)	2.03 (1.37, 3.01)
7. Coronary artery disease	195 (3.68)	107 (4.21)	88 (3.12)	0.47 (0.34, 0.66)
8. Allergic rhinitis	114 (2.69)	54 (2.74)	60 (2.63)	1.09 (0.70, 1.68)
9. Stroke	125 (2.63)	63 (2.67)	62 (2.58)	0.71 (0.46, 1.09)
10. Thyroid	112 (2.52)	17 (1.04)	95 (4.04)	4.49 (2.46, 8.18)
11. Depression	103 (2.01)	18 (0.79)	85 (3.28)	3.77 (2.07, 6.87)
12. Glaucoma	70 (1.43)	39 (1.91)	31 (0.93)	0.36 (0.21, 0.60)
13. Asthma	70 (1.38)	20 (0.89)	50 (1.89)	1.32 (0.66, 2.63)
14. Rheumatoid arthritis	64 (1.11)	5 (0.16)	59 (2.09)	10.71 (3.95, 29.02)
Comorbidity (2 or more diseases)^3^	1,952 (40.16)	757 (34.16)	1,195 (46.36)	1.22 (1.05, 1.42)

Values are presented as number (weighted %) or odds ratio (95% confidence interval).

1 The results were obtained taking into account the sampling design.

2 Adjusting for age and considering male as the reference group.

3 Comorbidity was defined as people with 2 or more of the 30 diseases.

**Table 4.** Results of evaluating the centrality of nodes in the comorbidity network of overweight and obesity in sexes over 45 years of age

Diseases	Overweight and obesity							
	Female				Male			
	Strength	Degree	Closeness	Betweenness	Strength	Degree	Closeness	Betweenness
Diabetes	4.50	3	0.20	0	4.84	3	0.45	3
Thyroid disease	1.71	1	0.17	0	-	-	-	-
Depression	1.91	1	0.16	0	-	-	-	-
Cataracts	13.46	5	0.20	11	-	-	-	-
Glaucoma	5.48	1	0.10	0	-	-	-	-
Asthma	12.61	2	0.11	11	-	-	-	-
Allergic rhinitis	7.18	1	0.06	0	-	-	-	-
Osteoarthritis	6.66	4	0.22	20	-	-	-	-
Rheumatoid arthritis	3.46	1	0.11	0	-	-	-	-
Osteoporosis arthritis	5.45	2	0.17	11	-	-	-	-
Hypertension	6.64	5	0.24	21	2.62	2	0.42	0
Dyslipidemia	9.49	6	0.23	21	5.45	3	0.44	3
Stroke	-	-	-	-	1.96	1	0.27	0
Coronary artery disease	11.43	4	0.21	20	2.39	1	0.25	0
